# Retroperitoneal paravertebral ganglioneuroma: a multidisciplinary approach facilitates less radical surgery

**DOI:** 10.1186/s12957-016-0953-y

**Published:** 2016-07-26

**Authors:** Christoph Paasch, Anja Harder, Esther Jasmin Gatzky, Ehssan Ghadamgahi, Andreas Spuler, Robert Siegel

**Affiliations:** 1 Department of General, Visceral and Cancer Surgery, HELIOS Klinikum Berlin-Buch, Berlin, Germany; 2 Faculty of Health, Witten/Herdecke University, Witten, Germany; 3 Institute of Pathology, Health Care Centre of the Municipal Hospital Brandenburg, Clinic of Medical University of Brandenburg, Brandenburg, Germany; 4 Institute of Neuropathology, University Hospital Münster, Münster, Germany; 5 Institute of Pathology, HELIOS Klinikum Berlin-Buch, Berlin, Germany; 6 Institute of Radiology, HELIOS Klinikum Berlin-Buch, Berlin, Germany; 7 Department of Neurosurgery, HELIOS Klinikum Berlin-Buch, Berlin, Germany

**Keywords:** Retroperitoneal tumour, Ganglioneuroma, Paravertebral tumour

## Abstract

**Background:**

Ganglioneuroma (GN) of the adult is a rare benign tumour originating from neural crest-derived cells. In most cases, GN is found in the mediastinum or retroperitoneum incidentally and may present with unspecific symptoms caused by space-occupying effects. The correct diagnosis of a retroperitoneal mass is still a challenge. Nevertheless, a preoperatively confirmed diagnosis of GN may support the concept of a less radical approach and may help to prevent unnecessary morbidity or loss of function.

**Case presentation:**

We report a case of a symptomatic retroperitoneal paravertebral GN in a 33-year-old woman. She has been referred with abdominal discomfort, lancinating pain in the right leg, headache and nausea. Magnetic resonance imaging revealed a solid paravertebral tumour adjacent to the psoas muscle. Computed tomography-guided core needle biopsy yielded the diagnosis of GN. The tumour was resected completely via a laparotomy. Immunohistopathological examinations confirmed a benign GN.

**Conclusions:**

Diagnostic studies and therapeutic interventions of retroperitoneal GN are discussed. In our case, a core needle biopsy preceding complete resection was helpful to prevent too extensive surgical approach.

## Background

Benign ganglioneuroma (GN) contains mature autonomic ganglion cells including satellite cells and long axonal processes as well as Schwann cells [1–3]. Those tumours originate from neural crest-derived cells which form the adrenal medulla and the sympathetic nervous system during embryonic development. Hence, they mainly arise in the mediastinum, retroperitoneum and pelvis where sympathetic ganglia are localized. Alike neuroblastoma and ganglioneuroblastoma, GN is supposed to represent the most mature neuroblastic tumour since ganglion cells that originate from neuroblasts are present in all of these tumours. Maturation of a neuroblastoma into a ganglioneuroma or ganglioneuroblastoma has been commonly described in the literature [1–3].

GN occurs in children and young adults, and median age at diagnosis is 7 years [4–7]. Distribution between males and females vary from a preference of the female gender [5] to no gender difference [7]. A higher frequency is reported in patients with multiple endocrine neoplasia type II and neurofibromatosis type 1. Due to their slow growth, most GNs are found incidentally. Most patients experience long-term disease-free survival even after incomplete resection [5, 7]. Malignant transformation is rare [8]. Since only a minority of GN are functional, distinct clinical symptoms are missing [4, 5]. Rarely, these tumours may produce hormones, such as catecholamines, vasointestinal peptides and androgens [7]. Radiographically, GN usually present as well-defined solid masses and do not contain cysts as opposed to neuroblastoma and ganglioneuroblastoma [6, 9]. Non-enhancement or slight enhancement in arterial phase and progressive mild enhancement in delayed phase is seen [6, 10]. At MR imaging, GN has low signal intensity on T1-weighted images and high signal intensity on T2 weighted images [6]. Distinct radiological features are lacking. Therefore, diagnosis can be challenging and is more precisely achieved by histological examination after resection. An imaging-guided core needle biopsy can be a reasonable approach to enable a reliable diagnosis before major surgery.

## Case presentation

**Fig. 1:**
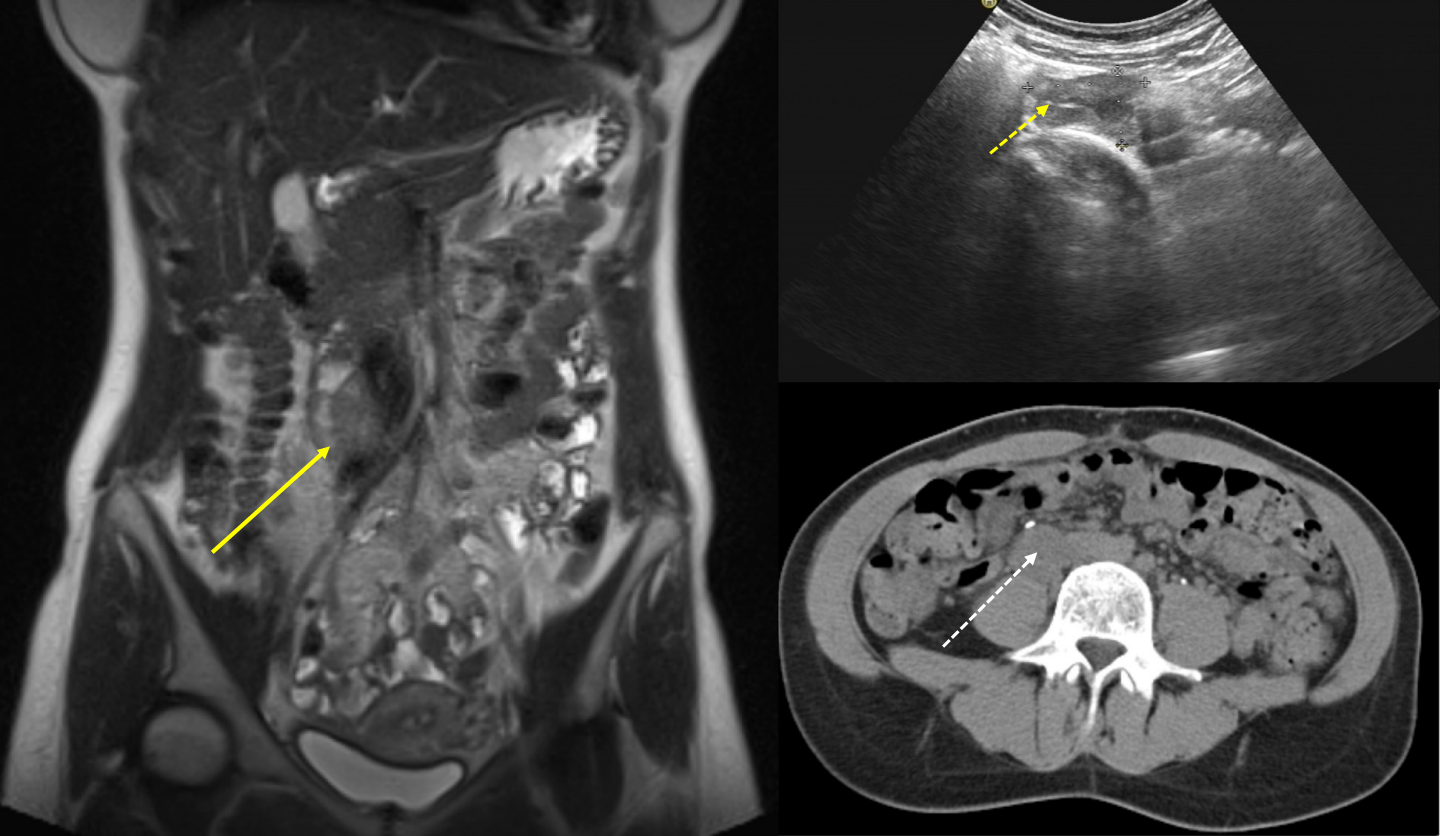
Preoperative coronal MRI showing a 68 × 35 × 28 mm solid mass anterior to the right psoas muscle (*yellow arrow*). The tumour was also detected by ultrasound and CT (*short yellow arrow*, *white arrow*)

**Fig. 2:**
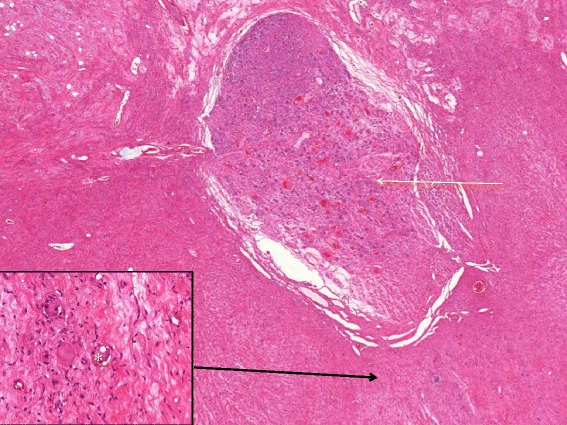
Representative part of the GN with a ganglion enclosed (*yellow arrow*, magnification ×2). Mature ganglion cells with small satellite cells are demonstrated in the insert (magnification ×20, haematoxylin-eosin)

A 33 year-old woman presented with a history of moderate abdominal pain and recurrent nausea. Recently, she experienced lancinating pain in her right leg and hot flashes. She did not report unintended weight loss, fatigue or fever. Previously, she had undergone body contouring plastic surgery after a weight loss of 35 kg. She carried a levonorgestrel-releasing intrauterine device (IUD) for several months. Computed tomography (CT) revealed a retroperitoneal mass adjacent to the right psoas muscle. Since a retroperitoneal sarcoma was suspected, the patient was referred to our institution. The laboratory test yielded normal blood levels of chromogranin A, neuron-specific enolase, CA15-3, and CA125. Urine and blood samples did not show elevated production of catecholamines, vasoactive intestinal peptide or androgens. Gynaecological examination including transvaginal ultrasound showed normal findings and confirmed the correct position of the IUD. Magnetic resonance imaging (MRI) demonstrated a solid tumour (68 × 35 × 28 mm) between the anterior circumference of the right psoas muscle and the anterior surface of the third and fourth lumbar vertebra as well as laterally extending to the to the right ureter, the inferior vena cava and right common iliac vein (Fig. 1). A CT-guided percutaneous core needle biopsy was performed to allow a histological diagnosis before surgery. The core biopsies contained only mature tissue with bundles of Schwann cells. Prominent, but also mature, ganglion cells were within the stroma, and there were no other histological signs of malignancy. Therefore, histopathological examination assumed a GN (Fig. 2).

Institutional interdisciplinary tumour board recommended a primary surgical resection. Prior to surgery, a double J stent was placed in the right ureter. Explorative laparotomy exposed the paravertebral tumour extending to the lower pole of the right kidney. The uterus and the adnexa with ovaries were without any pathological findings. After ureterolysis, the tumour was cautiously dissected from adherent structures such as the right common iliac vein and the kidney which were both not infiltrated by the tumour. Finally, the tumour was completely removed by means of blunt and sharp dissection and bipolar coagulation under a surgical microscope (the microscope has been used to save most of the branches of the lumbar plexus and nerves of the sympathetic trunk which was partly incorporated into the GN).

**Fig. 3:**
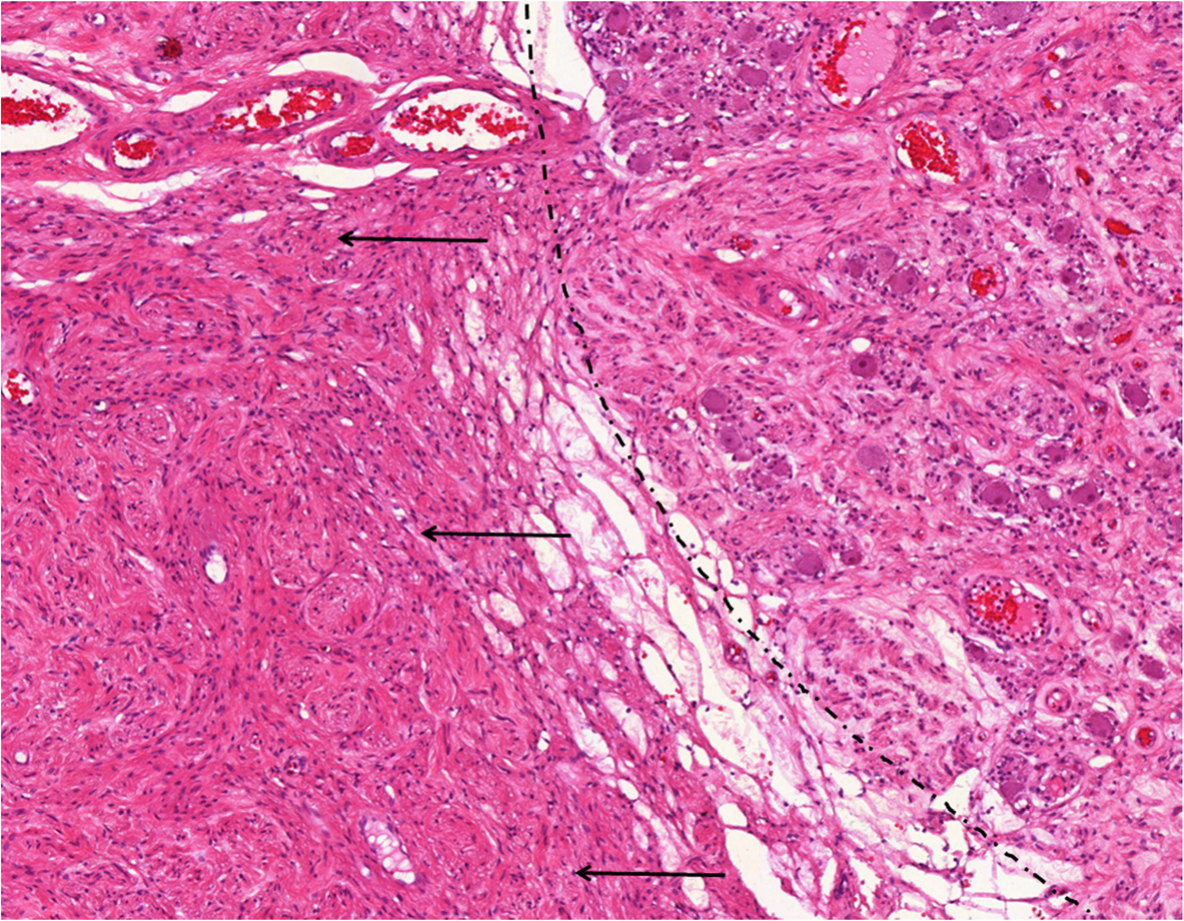
Tumour mass with a normal appearing ganglion (*right to dashed line*) merging into diffuse tumour areas (*left to dashed line*). Typically wavy cells with comma-shaped small nuclei prevail (haematoxylin-eosin, magnification ×10)

Neuropathological examination of the tumour established the diagnosis of a typical GN with extremely low proliferation (Ki67 less than 1 %) and no evidence of a ganglioneuroblastoma. Within the tumour, a ganglion with mature ganglion cells was embedded and a peripheral nerve was observed being penetrated by the tumour (Fig. 3).

The postoperative course was uneventful. After surgery, the patient reported heat sensations of stocking distribution in the right lower leg. Follow-up 4 months after surgery still revealed a small hyperthermic area and stocking-like heat sensations in the right lower leg that was interpreted to relate to a lesion of the sympathetic trunk partly incorporated into the GN. MRI at follow-up did not show tumour recurrence.

### Discussion

Clinical manifestations of GNs are diverse. In most cases, symptoms result from compression or displacement by the growing tumour and represent disturbances such as disruption of venous flow [11] and lower abdominal pain [12]. Most GNs are located mediastinal or retroperitoneal. In addition, a primary intraosseous GN of the sacrum has been described as a rare manifestation [13]. GN usually represents a non-secreting tumour. Occasionally, GN may release hormones that can cause diarrhoea, sweating and arterial hypertension [14]. Tumours may also cause tenesmuses and weight loss [15]. In our case, we additionally observed lancinating pain in a leg most likely caused by irritation of branches of the lumbar plexus running along the anterior surface of the psoas muscle.

Important differential diagnoses of retroperitoneal masses like the GN are retroperitoneal soft tissue sarcoma, and retroperitoneal fibrosis, and rarely lymphoma, a primary germ cell tumour, or metastatic testicular cancer.

The complete removal of the GN is described as adequate therapy with an excellent prognosis. Nevertheless, surgical morbidity has to be considered. In a retrospective series of 146 children with GN, surgical tumour resection resulted in an excellent long-term survival, but, nevertheless, 22 of the 146 patients (15 %) suffered surgery-related complications, of which two were fatal and seven were severe (e.g. Horner syndrome, rupture of thoracic aorta and thoracic haemorrhage) [5]. Residual GN after incomplete resection remains stable for years without malignant transformation [5]. In addition, Retrosi et al. did not observe recurrences after incomplete excision in a series of 23 children with thoracic (*n* = 14), abdominal (*n* = 7) or pelvic GN (*n* = 3) [16]. Surgical complication rate reached 30 % [16]. Therefore, Retrosi and De Bernadi recommended less radical surgery in childhood GN to reduce surgery-related morbidity and mortality [5, 16]. Even a watchful waiting could be an option in selected cases after secured diagnosis. Interdisciplinary discussion and image-guided biopsy prior to surgery is recommended to prevent unnecessary expanded surgical approaches with significant postoperative morbidity. From our experience, we would suggest surgical resection of GN, but with a less radical approach in order to preserve the adjacent organs and structures where appropriate.

## Conclusions

Ganglioneuroma is a benign, rare tumour with an excellent prognosis in long-term survival even after incomplete resection. Therefore, a limited surgical resection to prevent postoperative morbidity might be appropriate in selected cases. An image-guided biopsy prior to surgery can establish a histological diagnosis and prevent unneeded extensive surgery.

## Abbreviations

CT, computed tomography; GN, ganglioneuroma; IUD, intrauterine device; MRI, magnetic resonance imaging
